# Heart rate responses provide an objective evaluation of human disturbance stimuli in breeding birds

**DOI:** 10.1093/conphys/cot013

**Published:** 2013-06-21

**Authors:** Ursula Ellenberg, Thomas Mattern, Philip J. Seddon

**Affiliations:** Department of Zoology, University of Otago, 340 Great King Street, PO Box 56, Dunedin, New Zealand

**Keywords:** Heart rate telemetry, human disturbance, stress-coping styles, tourism impact, wildlife management, Yellow-eyed penguins

## Abstract

Intuition is a poor guide for evaluating the effects of human disturbance on wildlife. Using the endangered Yellow-eyed penguin, *Megadyptes antipodes*, as an example, we show that heart rate responses provide an objective tool to evaluate human disturbance stimuli and encourage the wider use of this simple and low-impact approach. Yellow-eyed penguins are a flagship species for New Zealand's wildlife tourism; however, unregulated visitor access has recently been associated with reduced breeding success and lower first year survival. We measured heart rate responses of Yellow-eyed penguins via artificial eggs to evaluate a range of human stimuli regularly occurring at their breeding sites. We found the duration of a stimulus to be the most important factor, with elevated heart rate being sustained while a person remained within sight. Human activity was the next important component; a simulated wildlife photographer, crawling slowly around during his stay, elicited a significantly higher heart rate response than an entirely motionless human spending the same time at the same distance. Stimuli we subjectively might perceive as low impact, such as the careful approach of a ‘wildlife photographer’, resulted in a stronger response than a routine nest-check that involved lifting a bird up to view nest contents. A single, slow-moving human spending 20 min within 2 m from the nest may provoke a response comparable to that of 10 min handling a bird for logger deployment. To reduce cumulative impact of disturbance, any human presence in the proximity of Yellow-eyed penguins needs to be kept at a minimum. Our results highlight the need for objective quantification of the effects of human disturbance in order to provide a sound basis for guidelines to manage human activity around breeding birds.

## Abstract (precis)

What humans may consider to be a careful approach can constitute a significant disturbance event for some species. Routine checks to view nest contents affect Yellow-eyed penguins less than prolonged observation from a distance. Evaluating disturbance stimuli via heart rate telemetry provides a reliable basis for effective visitor management.

## Introduction

Iconic species such as the Yellow-eyed penguin, *Megadyptes antipodes*, which are rare, endemic, and endangered (BirdLife International, 2012), are key draw-cards for nature-based tourism in southern New Zealand. However, Yellow-eyed penguins exposed to unregulated visitor access show significantly reduced breeding success (Ellenberg *et al.*, 2007) and fledge chicks at lower weights, which subsequently reduces first year survival (McClung *et al.*, 2004; Ellenberg *et al.*, 2007).

As the growth of nature-based tourism is expected to continue unabated, it is important not only for ecological but also for economic sustainability to minimize associated human impacts (Seddon and Ellenberg, 2008). Well-managed visitation of even rare and endangered wildlife can be positive for conservation. However, appropriate species- and site-specific management decisions require rigorous research to understand the nature of disturbance-related impacts.

A standard approach to evaluate human disturbance stimuli is the monitoring of behavioural responses in animals. For example, the distance at which an animal reacts to human presence can be quantified experimentally and findings used to define minimal approach distances (e.g. Rodgers and Smith, 1995; Giese, 1998; Carney and Sydeman, 1999; Rodgers and Schwikert, 2002). However, behavioural responses can be an unreliable predictor of the real impact of human disturbance (Gill *et al.*, 2001; Fernández-Juricic *et al.*, 2005; Tarlow and Blumstein, 2007). Although ethology can contribute to the better management and conservation of species (Buchholz, 2007; Caro, 2007), overt behavioural reactions, or lack of them, are a poor guide to the impact of disturbance caused by humans.

Animals may perceive humans as potential predators, and the risk-disturbance hypothesis makes predictions about associated effects (Gill and Sutherland, 2000; Frid and Dill, 2002). Similar to antipredator responses, reaction to human disturbance can affect individual fitness via the energetic and lost-opportunity costs of risk avoidance (Frid and Dill, 2002). The distance at which an animal will tolerate human proximity varies with the type of disturbance, species, and intraspecifically according to character, age, condition, current behaviour, availability of alternative habitat, time of day, stage of breeding, and previous experiences (Gill *et al.*, 2001; Seddon and Ellenberg, 2008). Early in the breeding season, human disturbance not only caused egg loss and nest abandonment but also disrupted recruitment of pre-breeding birds (Hockey and Hallinan, 1981; Woehler *et al.*, 1994). Once nests are established, most penguin species show little behavioural reaction to human presence (Culik and Wilson, 1991; Wilson *et al.*, 1991; Nimon *et al.*, 1995; UE, TM, and PJS, personal observations), which is often mistaken for habituation (see Seddon and Ellenberg, 2008).

Measuring the heart rate (HR) responses has been used to evaluate single disturbance events (e.g. Culik *et al.*, 1990; Chabot, 1991; Neebe and Hüppop, 1994; Hüppop, 1995; Holmes *et al.*, 2005; de Villiers *et al.*, 2006; Ellenberg *et al.*, 2012). For example, incubating Humboldt penguins, *Spheniscus humboldti*, show little change in behaviour when being approached by a person (Ellenberg *et al.*, 2006); however, the maximal heart rates measured during human approach are comparable to those reached while running (Butler and Woakes, 1984).

In recent years, HR has been used to estimate the energy expenditure of free-ranging animals (e.g. Storch *et al.*, 1999; Bevan *et al.*, 2002; Green *et al.*, 2005; Grémillet *et al.*, 2005). Heart rate during physical exertion on land is linearly correlated with the rate of oxygen consumption, hence metabolic rate, in several penguin species (Bevan *et al.*, 1995; Froget *et al.*, 2001; Green *et al.*, 2001). This linear relationship between HR and metabolic rate holds true for resting penguins exposed to a range of ambient temperatures (Froget *et al.*, 2002). Changes in basal field resting heart rates (RHR) as a result of different disturbance regimens can help quantify energy budgets (Halsey *et al.*, 2008) and evaluate potential chronic stress (Ropert-Coudert *et al.*, 2009). Not only exercise or ambient temperature but also ‘emotional’ stimuli, such as slamming the door to an experimental room, may increase the HR and the correlated energy expenditure (Cyr *et al.*, 2008). Hence, HR measured during stressful events allows the assessment of relative costs associated with different stimuli.

Additional energetic demands on breeding birds are of concern, because even subtle costs of human disturbance can accumulate (Moberg, 2000; Teixeira *et al.*, 2007) and may ultimately lead to population-level consequences (Seddon and Ellenberg, 2008). To evaluate the range of disturbance stimuli to which breeding penguins are regularly exposed, we measured the HR responses of individual birds during natural behaviour and experimental human disturbance. We aimed to determine the most important factors affecting responses and compared the relative energetic costs of different disturbance stimuli, using HR as a proxy, so as to provide a basis for effective and anticipatory management decisions.

## Methods

### Study site and species

The Otago Peninsula on the east coast of New Zealand's South Island is one of the most important mainland breeding sites for Yellow-eyed penguins. Dunedin, the gateway to the Peninsula, promotes itself as ‘New Zealand's Wildlife Capital’, and provides opportunities for guided tours as well as information about where local wildlife can be viewed free of charge. Since the early 1990s the numbers of visitors have increased by more than an order of magnitude, and concern has been expressed by a number of people and agencies that tourism-related pressures may be becoming too great (Seddon *et al.*, 2003).

During the austral summer breeding season 2005–2006 we recorded the behaviour and HR response of incubating Yellow-eyed penguins to a variety of natural and experimental stimuli. We studied birds breeding at tourist-exposed Sandfly Bay (45°89′S, 170°64′E) and the neighbouring, less-disturbed Boulder Beach complex (45°53′S, 170°37′E) on the Otago Peninsula. Both sites have been monitored by the New Zealand Department of Conservation since 1981. Individuals are usually marked as fledglings, using metal flipper bands. Unknown adults are banded when attending a nest in the area. For all focal birds, the sex was known (either from blood tests or from morphometric measurements; Setiawan *et al.*, 2004). We found the factors sex, character and previous bleeding experience (bled) to have a significant influence on both the initial stress response and the habituation potential of individuals (Ellenberg *et al.*, 2009). Hence, all three factors were considered when testing for differences in HR response to human disturbance experiments.

Unlike other penguin species, which usually nest in colonies, Yellow-eyed penguins are solitary breeders. They nest under dense vegetation, mostly obscured from the open, and visually isolated from each other (Darby and Seddon, 1990). The breeding habitat at our study sites allowed us to work with each individual penguin separately, so that only the bird attending the focal nest could see and hear the approaching person.

A total of 18 nests were included in the study, resulting in data for 34 individuals at three locations: Double Bay (11) and Midsection (15), both in the Boulder Beach complex, and tourist-exposed Sandfly Bay (8). All experiments were ­performed silently by a single person (UE), dressed in inconspicuous clothing, moving in a calm, steady manner (i.e. slow and even steps, <1/s; no quick movements). Experiments included typical human encounters experienced by nesting Yellow-eyed penguins on the Otago Peninsula, as well as one standardized disturbance experiment (2 m-stop). Additionally, we recorded HR of seven guard-stage males during handling for logger deployment on Whenua Hou (Codfish Island, 46°78′S, 177°99′E).

### Heart-rate telemetry and hidden cameras

The HR of incubating penguins was recorded via an egg-shaped dummy (ED) added to the clutch. This is the least-intrusive method currently available to determine HR responses in birds (Nimon *et al.*, 1996; Giese *et al.*, 1999; Tarlow and Blumstein, 2007). The ED contained an internal omni-directional lavalier condenser microphone (WL183, Shure Inc., adapted by Strawberry Sound, Dunedin, New Zealand, to suit field conditions). The HR signal was transmitted up to 100 m via UHF (ULXP series, Shure Inc.) to a mobile hide, where the receiver station was situated. The HR signal was recorded to MiniDisc (Sharp MD-MT88). Simultaneously, we observed behavioural changes, natural stimuli (e.g. approaching partner or neighbour), and disturbance experiment details via previously deployed generic weatherproof surveillance cameras. The video signal was transmitted to the hide using a 100 m high-quality coaxial TV cable.

We deployed the ED when only one partner was present. During ED deployment, the personality trait or character of each bird was classified as follows: timid, bird backed off nest ready to flee or abandoned nest during human approach; frozen, bird remained static on the nest, observed the approaching person but did not show any aggressive reaction to being touched at the chest and lifted up slightly from the nest to allow ED placement; or aggressive, bird responded with pecks or flipper beats when the person reached the nest, on occasion even charging towards the experimenter (Ellenberg *et al.*, 2009).

Following a pilot study testing ED acceptance in Yellow-eyed penguins over a 24 h period, we reduced the ED deployment time to 4–10 h in order to eliminate any risk of compromising reproductive success. On two occasions we decided to leave the ED in over night (31.5 and 32 h) and were able to obtain HR data of partners that had not experienced ED deployment. These two ‘naïve’ individuals responded comparably to all other birds [HR increase (%RHR; see below), *t*_28_ = 0.433, *P* = 0.668; recovery time, *t*_25_ = −0.416, *P* = 0.681; 2 m-stop experiment, HR maximum 139 and 151 beats/min, average HR level maintained during human proximity 116 ± 7 and 130 ± 4 beats/min, and recovery time 688 and 722 s; compare Table 2]. Likewise, previous work on Humboldt penguins did not find any differences in HR response to experimental disturbance between essentially naïve birds and their mates that experienced ED deployment (Ellenberg *et al.*, 2006). From these results, we conclude that adding an ED to the clutch at least 1 h prior to disturbance experiments did not alter subsequent HR response significantly.

**Figure 1:**
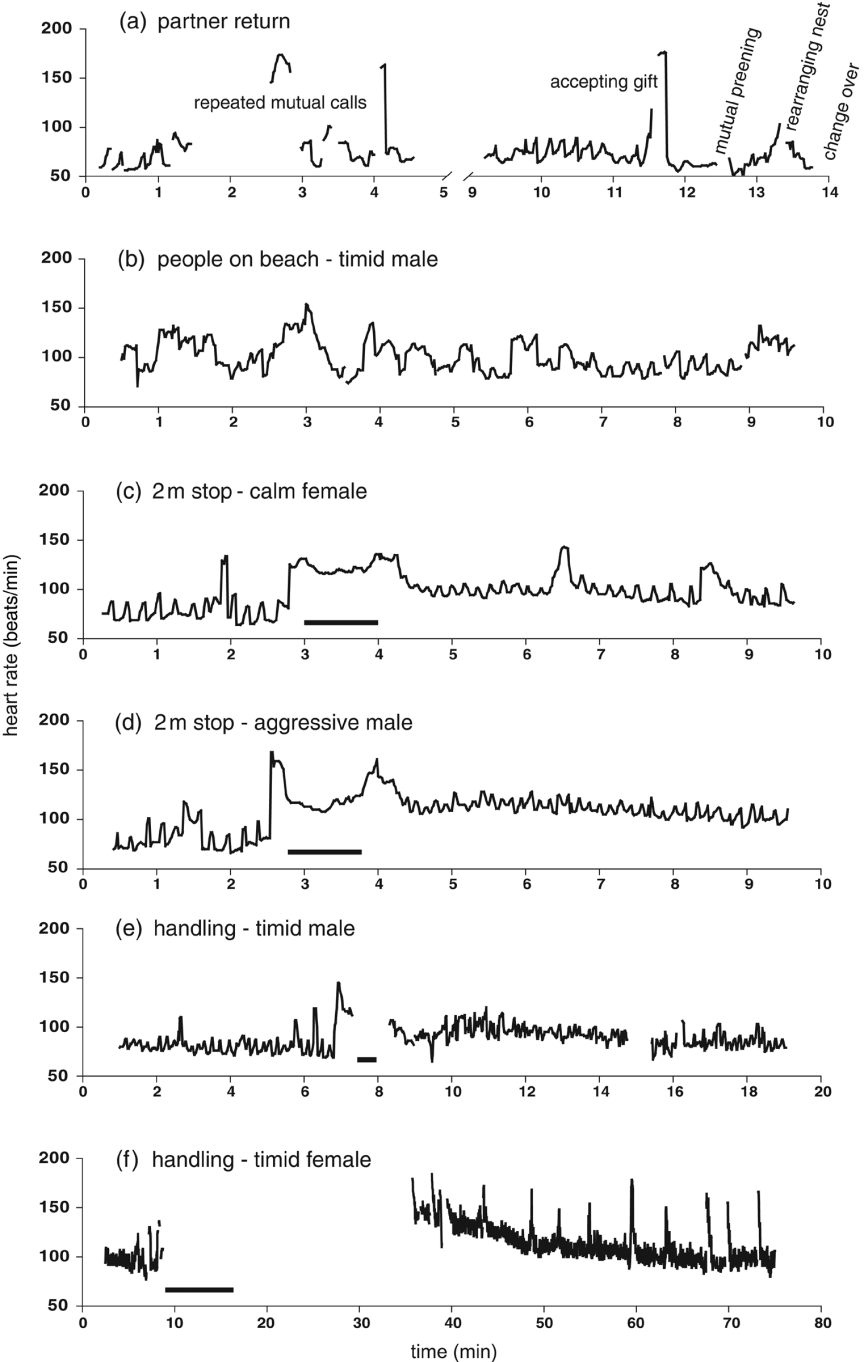
Examples of Yellow-eyed penguin heart rate response (in beats per minute) during natural stimuli and human disturbance. (**a**) Partner return and pair interaction. (**b**) Half an hour after three visitors had settled at 10 m distance, out of sight. (**c–f**) Disturbance experiments: human approach (c and d); and capture and handling (e and f). Bars indicate the 1 min motionless stop at 2 m from the nest (c and d) or handling time (e and f). Gaps in the curves are a result of a temporary loss of the heart rate signal due to absence from the clutch or mutual calls.

Sound data were analysed with custom-written software in Matlab 6.5 (Mathworks, Natick, MA, USA). Essentially, a power-spectral-density analysis via double fast Fourier transform resulted in peaks that corresponded to HR (in beats per minute). Data were manually validated, and peaks with small margins (<10) were usually retained in the analysis. As the HR signal is strongest in low frequencies (100–300 Hz), we used a microphone potent for base frequencies; hence, most ambient noise, such as that created by moving the ED, generally did not interfere with the HR signal. Sound quality was further improved by including a small lead weight in the base of the ED so that the microphone was always pointing towards the brood patch. Even high ambient noise levels, such as those created by a passing aeroplane (400–1000 Hz), can be eliminated easily. However, mutual calls will obscure the HR signal (Fig. 1a). The HR signal is lost as soon as a bird gets up from the clutch.

As for Humboldt penguins (Ellenberg *et al.*, 2006), Yellow-eyed penguin resting heart rate (RHR) was found to be as low as 50 beats/min, thus we calculated moving averages using an interval of 12 s (containing a minimum of 10 heart beats) for each heart rate reading. Following Neebe and Hüppop (1994), we defined baseline as the mean RHR during a period of at least 30 s of undisturbed incubation immediately before the experiment or natural stimulus; two standard deviations from mean RHR were considered a tolerance band. When the HR left the tolerance band spontaneously, it counted as excitation. Maximal HR increase considered sound data from 12 s around the peak HR response. Average HR during human proximity included 60 HR readings obtained during the 1 min stay at the nest. The excitation ended when the HR was maintained for at least 30 s within the previously defined tolerance band. Recovery time was defined as the time from when the person simulating a disturbance event turned back to retreat out of sight to the end of excitation. Recovery time was independent of RHR (linear regression, *F*_1,26_ = 0.063, *P* = 0.805, *r*^2^ = 0.002) and HR increase (*F*_1,26_ = 1.767, *P* = 0.196, *r*^2^ = 0.066); hence, we analysed HR increase and recovery time separately. As RHR may vary depending on nest microclimate and other factors, we compared levels of excitation using the relative HR increase (%RHR).

### Natural stimuli

We recorded potential natural stimuli, such as a calling neighbour or a returning partner, and the associated behaviour of the incubating bird (e.g. alert look, call), as well as natural behaviours, such as nest maintenance or preening (for detailed descriptions of behaviours mentioned here and later in the text see Richdale, 1951). In all cases, we analysed the maximal HR increase and recovery time. If we were able to record several natural events of one type for the same bird, individual means were used.

### Disturbance experiments

Table 1 lists disturbance experiments performed, as well as the natural maintenance stimulus that was used as a baseline measure. Recordings were analysed with regard to maximal HR increase, average HR during the stay of a person at 2 m distance from the nest (where applicable), and time needed for recovery. If several disturbance experiments were performed on the same bird, we waited at least 1 h following the experiment to ensure that the HR had long returned to previously measured RHR levels before the next experiment started.

**Table 1:** #Definitions of natural and experimental stimuli that resulted in Yellow-eyed penguin heart rate responses

Stimulus	Definition
Maintenance	Includes both preening and nest maintenance behaviours
2 m-stop	First ever measured direct approach of the researcher to the incubating penguin, stopping motionlessly at 2 m distance from the nest for 1 min within sight of the bird before retreating out of sight
Photographer	Simulated wildlife photographer, similar to 2 m-stop experiment; however, the 1 min stay was used to move about carefully on the belly at 2 m distance, avoiding quick movements; given that the movements of the experimenter were restricted by dense vegetation, the original position was shifted by less than a metre during the process; once the bird was reasonably visible in the frame, a photograph was taken. One minute at the nest is usually too short a time frame to take a good picture, but we decided to stick to the initial experimental protocol (i.e. 1 min at 2 m from the nest)
Nest-check	Direct approach and touching the incubating bird at the chest, lifting it up slightly to get a swift look at nest contents. The time spent at under 2 m distance from the bird was about 30 s. Flighty birds that retreated slightly upon approach and exposed the nest contents were nevertheless touched at the chest to maintain experimental consistency
Handling (logger)	Direct approach to the bird, capture and restraint for logger deployment (for method compare Wilson *et al.*, 1997); heart rate was measured immediately after capture (maximal heart rate), after taking body measurements, and during dive data logger deployment, while the birds usually stayed motionless, with their heads covered using a loose brown cotton bag; no heart rate reading was obtained prior to and after handling
Handling (bands)	Direct approach to the incubating bird, capture, and restraint at the nest site for banding and band-fixing purposes; heart rate reading was obtained prior to and after, but not during handling. Following handling, the bird was released at about 2 m distance from the clutch, facing the nest; after release, the experimenter retreated immediately out of sight and back to the mobile hide

The inclusion of a standardized experimental pass of the nest was not possible due to topography and densely vegetated habitat.

### Relative intensity of human disturbance stimuli

Responses were quantified via the integral of HR level increases over time. Natural maintenance behaviour (i.e. preening and nest maintenance) was used as a point of ­reference to compare different stimuli. The integral of HR changes resulting from disturbance stimuli is given as multiples of the integral of HR changes during natural maintenance behaviour. The relative effect was conservatively estimated. Firstly, as reference, we used the peak maximal HR measured during a typical 20 s of maintenance behaviour; hence, baseline costs are overestimated. Secondly, we ignored the usually short-lived peak maximal HR during human disturbance but integrated a simple curve of average HR during human proximity and the time needed for recovery. For the estimate of relative energy expenditure during ‘handling’, we used HR obtained during logger deployment combined with recovery times measured following band-fixing. We did not consider periods of increased vigilance and displacement behaviours following human disturbance events. Therefore, the severity of human disturbance stimuli is likely to be underestimated.

### Statistical analysis

We calculated the circular–linear correlation (Berens, 2009) to test for potential effects of daytime on HR responses. The possible effects of research manipulations on hatching success were tested using a two-tailed *t*-test comparing independent means. A paired *t*-test was used to compare HR responses of individual penguins during natural stimuli and standardized human disturbance (2 m-stop experiment). We employed repeated-measures analysis of variance (ANOVA) to test for differences in HR response within and between subjects when comparing several disturbance experiments. Following significant ANOVA results, we used Tukey–Kramer honestly significant difference as our criterion for significance (Zar, 1999). We square-root-transformed (sqrt) data when assumptions of normality were not met, and we tested for homogeneity of variances using Levene's test. Pearson's correlation coefficient was calculated to quantify correlation. We considered differences to be significant if *P* < 0.05 and reported values as means ± SD, unless indicated otherwise.

## Results

### Effect of research on productivity

Hatching success was similar in nests exposed to disturbance experiments compared with control nests in the same area (*t*_39_ = −1.24, *P* = 0.223). We conclude that short-term ED and hidden camera deployment, as well as a few short and careful disturbance experiments, had no effect on the productivity of study birds.

### Natural stimuli

Resting heart rates prior to a stimulus were on average 77 ± 12 beats/min (*n* = 106) and similar among all groups. Partner return and associated behaviours provoked the strongest HR increase observed during natural stimuli. The maximal HR measured was 174 beats/min (282%RHR) during mutual calls and 177 beats/min (287%RHR) when accepting a gift of nesting material (one observation; Fig. 1a). In all cases, the HR dropped back to RHR levels within seconds after the stimulus ended (maximal recovery time observed was 18 s during partner return after the first set of mutual calls). The recovery from heart rate increase caused by natural stimuli rarely took more than 1 min. The time allocated to nest maintenance or preening behaviour was usually short, but even in one case of extended nest sorting (>5 min in three spells) the bird recovered within 23 s after it had settled back on the clutch. Preening and nest maintenance caused similar HR responses (HR increase, *t*_25_ = −0.81, *P* = 0.425; and recovery time, *t*_21_ = 0.71, *P* = 0.486); hence, these were combined as ‘maintenance’ to increase the sample size for subsequent analysis. The excitation during maintenance behaviour was unrelated to the time spent rearranging nest contents or preening, and caused considerably lower HR responses than partner return (see above; 158 ± 13%RHR, 45 ± 56 s recovery time). Maintenance behaviour elicited similar responses in tourism and control areas (HR increase, *t*_25_ = −0.004, *P* = 0.997; and recovery time, *t*_5.8_ = 0.17, *P* = 0.875). Heart rate responses as a result of calling by neighbouring pairs (*n* = 3) or the sight of a passing juvenile (*n* = 1) were ­comparable to those observed during natural maintenance ­behaviour.

### Human stimuli

Human stimuli usually caused greater HR increases than natural stimuli and always provoked substantially longer recovery times. An experimental approach to within 2 m from the nest site caused significantly greater individual HR responses than maintenance behaviour (HR increase, 196 ± 24%RHR, paired *t*-test, *t*_18_ = 6.02, *P* < 0.001; and recovery time, 477 ± 328 s, paired *t*-test (sqrt recovery time), *t*_16_ = 7.013, *P* < 0.001; even though RHR were similar, 77 beats/min, paired *t*-test: *t*_19_ = −1.14, *P* = 0.270). In all experiments, elevated HR was maintained, and the birds did not recover during human presence. In one instance, three tourists settled on the beach about 10 m from a penguin nest that was equipped with an ED. The tourists were out of sight of the bird and oblivious of its presence, talking to each other for more than 30 min. Despite being only an acoustic stimulus, the bird's HR remained elevated throughout their presence. The HR varied with discussion patterns of the group, i.e. higher pitched and louder voices provoked HR increase, whereas pauses in the conservation allowed for temporary recovery (compare Fig. 1b). For disturbance experiments, the maximal HR was recorded during a nest-check (189 beats/min) and the maximal recovery time observed was almost an hour (57.2 min) after a 3.2 min handling procedure that involved fixing a flipper band.

The date or time of day when the experiment was performed had no effect on RHR [circular–linear regression (daytime), ρ_cl_ = 0.28, *P* = 0.279; and linear regression (date), *F*_1,31_ = 2.25, *P* = 0.144], HR increase (daytime, ρ_cl_ = 0.26, *P* = 0.326; and date *F*_1,30_ = 0.26, *P* = 0.614), or recovery time (daytime, ρ_cl_ = 0.15, *P* = 708; and date, *F*_1,29_ = 0.05, *P* = 0.829). Cloud coverage or wind speed during the experiment was equally unimportant (HR increase, *F*_1,30_ = 0.09, *P* = 0.772; and recovery time, *F*_1,29_ = 0.04, *P* = 0.842).

Following disturbance experiments, after having recovered by definition (see Methods section) the birds usually remained vigilant, and the ‘RHR’ was regularly interrupted by alert look events (Fig. 1f). Nest maintenance activity within 30 min after defined recovery caused greater HR increase (177 ± 12%RHR) and was followed by significantly longer recovery times (maximum 7 min, unpaired *t*-test, HR increase in %RHR, *t*_19_ = −3.88, *P* = 0.001; and recovery time *t*_3.23_ = −3.31, *P* = 0.004) than nest maintenance under natural circumstances.

### Comparing human disturbance stimuli

During the 1 min that the experimenter spent at 2 m from the nest, the birds maintained their average HR at significantly higher levels when the experimenter was carefully moving about, mimicking a wildlife photographer (‘photographer’ experiment), compared with staying motionless [‘2 m-stop’, repeated-measures ANOVA within-subjects factor average HR increase (%RHR), *F*_1,13_ = 5.26, *P* = 0.039], whereas the peak maximal HR and time needed for recovery did not ­differ.

Likewise, the maximal HR increase and recovery time did not differ significantly when comparing individual HR responses during three different experiments [2 m-stop, ­photographer, and nest-check; *n* = 10, repeated-measures ANOVA, within-subjects factor ‘experiment’, HR increase (%RHR), *F*_2,18_ = 1.77, *P* = 0.199; and recovery time (sqrt), *F*_2,16_ = 0.70, *P* = 0.513]. Here, location was an important between-subjects factor, with birds exposed to unregulated tourism responding more strongly than birds at neighbouring less-disturbed sites [HR increase (%RHR), *F*_1,9_ = 4.22, *P* = 0.070; and recovery time (sqrt), *F*_1,8_ = 8.50, *P* = 0.019]. Field RHR did not differ between nests exposed to unregulated tourism and nests in neighbouring control areas (*t*_30.1_ = 0.52, *P* = 0.611). Considering sex and character as potential factors affecting RHR, we still could not detect any difference in RHR of birds exposed to different disturbance regimens (*F*_1,32_ = 0.08, *P* = 0.776).

Approaching a bird to within 2 m of the nest site (2 m-stop) provoked a similar HR response as did a nest-check that involved touching the bird on the chest (repeated-measures ANOVA, within-subjects effect HR increase (%RHR), best model included factor sex, *F*_1,16_ = 1.10, *P* = 0.320; and recovery time (sqrt), best model included factors bled and character, *F*_1,11_ = 0.004, *P* = 0.951). Females reached significantly lower maximal HR values than males (between-subjects factor sex, *F*_1,16_ = 5.30, *P* = 0.035; compare Fig. 1c and d). The factors character and previous bleeding experience, as well as the interaction term of both, were important predictors for interindividual differences in recovery time (sqrt, between-subjects effects, bled, *F*_1,11_ = 5.80, *P* = 0.035; character, *F*_1,11_ = 4.64, *P* = 0.035; and bled × character, *F*_1,11_ = 5.13, *P* = 0.045); i.e. non-bled birds and aggressive individuals needed less time to recover. This relationship remained the same when testing sexes separately.

Capture and handling clearly provoked the longest recovery times [paired *t*-test, recovery time (sqrt), *t*_4_ = −7.47, *P* = 0.002]. Recovery time was independent of handling time. Again, we observed considerable individual differences; for example, a flighty male used to being handled recovered within 9:59 min (Fig. 1e), whereas, a naïve flighty female needed more than 15 min to return to its clutch after being handled, and even after having recovered by definition (42.1 min) the bird remained vigilant, and heart rate ­continued to be frequently interrupted by alert look events with associated HR peaks (Fig. 1f).

Table 2 gives an overview of differences in HR responses to a range of stimuli.

**Table 2:** Yellow-eyed penguin heart rate responses to natural and human-derived stimuli

Stimulus	RHR^a^ (beats/min)	Maximal HR increase (beats/min)		Average HR increase, during 1 min (beats/min)		Recovery time (s)	
Maintenance	77 ± 10 (28)	119 ± 17 (27)	**A**	—	–	40 ± 50 (23)	**A**
2 m-stop	77 ± 12 (33)	148 ± 18 (32)	**B**	114 ± 16 (31)	**B**	609 ± 413 (31)	**B**
‘Photographer’	81 ± 13 (15)	150 ± 19 (15)	**B**	125 ± 16 (15)	**C**	548 ± 515 (15)	**B**
Nest-check	75 ± 12 (25)	146 ± 19 (25)	**B**	—	–	591 ± 297 (22)	**B**
Handling (logger)^b^	—	129 ± 20 (9)	*	107 ± 18 (8)	**B**	—	–
Handling (bands)^c^	87 ± 9 (5)	156 ± 20 (5)	**B**	—	–	2070 ± 1160 (5)	**C**

Data are given as means ± SD (number of individuals). Note that this is an overview of all results, while statistical tests had paired or repeated-measures designs to account for individual differences (for details refer to main text). Bold letters group similar responses within each measured parameter; different letters indicate significant differences. Maximal and average heart rate (HR) increase are given in beats per minute. Recovery time is given in seconds. For definitions of stimuli see Table 1.

^a^Resting heart rates (RHR) prior to a stimulus were on average 77 ± 12 beats/min (*n* = 106) and similar among groups.

^b^Heart rate reading during logger deployment only.

^c^Heart rate reading prior to and after, but not during handling.

*Excluded from statistical analysis because maximal HR was reached prior to capture and restraint.

### Relative effect of human stimuli

**Figure 2:**
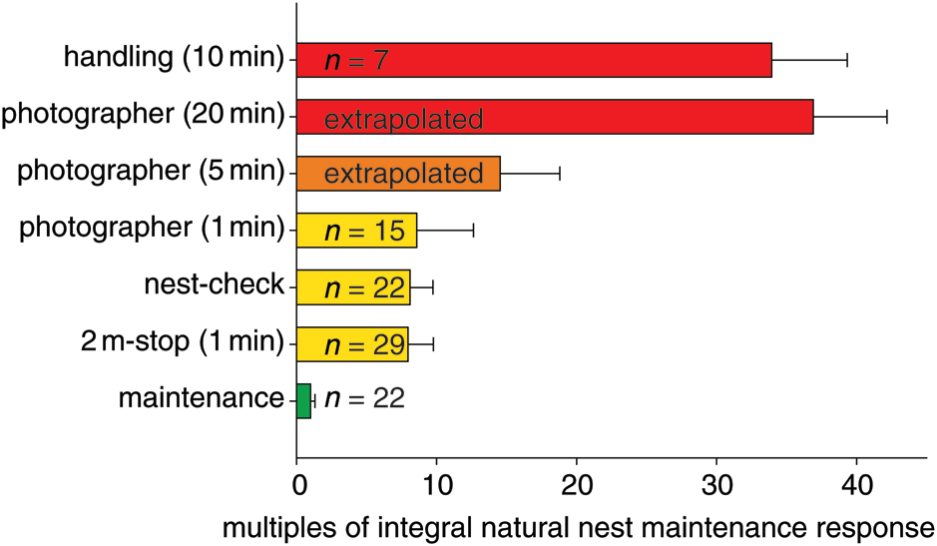
Relative severity of a range of human disturbance stimuli (compare Table 1) given as multiples of the integral heart rate response during natural nest maintenance. Error bars represent 95% confidence intervals. Different colours indicate significant differences in responses following Tukey's honestly significant difference test (*F*_1,125_ = 90.83, *P* < 0.001).

Handling a bird for 10 min provoked an at least 34 times higher HR response than did the average preening or nest maintenance event. The response to a routine nest-check was eight times higher than that to maintenance and comparable to a motionless stop of 1 min at 2 m distance from the nest. Moving slowly about on the belly for the same time at the same distance (‘photographer’) provoked a slightly higher response, on average nine times that of maintenance behaviour. As we never observed recovery so long as a person remained within sight of a Yellow-eyed penguin, we extrapolated the integral of HR response for a wildlife photographer staying 5 or 20 min at close proximity to the nest. Figure 2 gives an overview of the relative severity of a range of human disturbance stimuli to which Yellow-eyed penguins are regularly exposed at their breeding sites.

**Figure 3:**
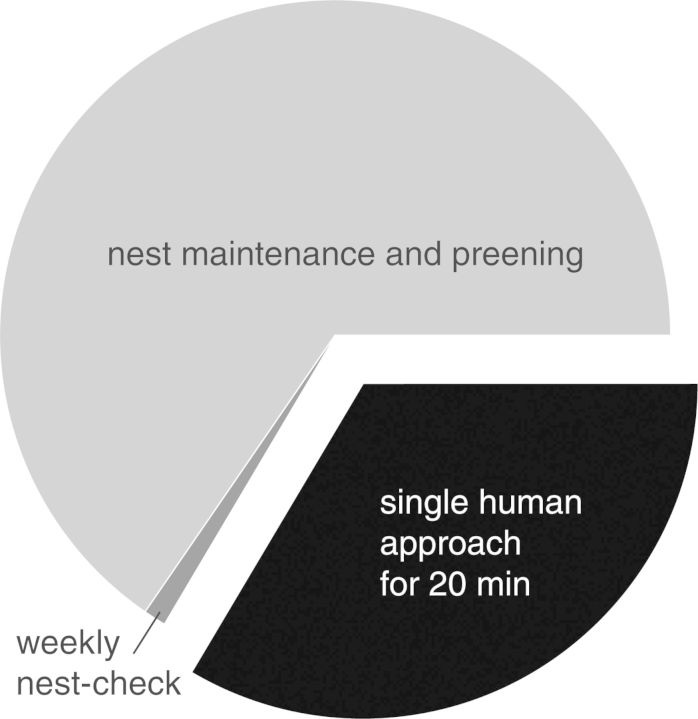
Estimated energy expenditure caused by a single careful human approach (‘photographer’) for 20 min and the weekly nest-check in relationship to a typical daily energy budget of an incubating Yellow-eyed penguin.

Figure 3 provides an assessment of the influence of human activity on a typical daily energy budget for Yellow-eyed penguins during incubation, using the integral of HR response to different stimuli as proxy for energetic costs. From observations, we know that Yellow-eyed penguins perform an average of three nest maintenance or preening events per hour, accumulating to 72 such events during a day. As Yellow-eyed penguins are solitary breeders and spent on average 2 days on their clutch during each incubation shift, we have ignored commuting and socializing behaviours. Adult Yellow-eyed penguins, even at intensely studied sites, get handled less than once a year. While not all nests are easily accessible to humans, those that are get regularly approached by under-regulated tourists, often leading to nest failure.

## Discussion

Yellow-eyed penguin maintenance behaviours or natural stimuli can cause HR peaks comparable to those measured during human disturbance; however, the time needed to recover after natural excitation was short in comparison to the long recovery times following human disturbance. Human proximity was the most important factor for predicting HR responses to human disturbance stimuli, with no sign of recovery as long as a person was within sight. Human behaviour was also important; a person carefully moving around on their belly provoked a significantly greater HR response than did a motionless human at the same distance. Likewise, in King penguins (*Aptenodytes patagonicus*) HR responses during agnostic encounters depended on the intensity and duration of the stimulus, with the latter being the more important factor in motionless bystanders witnessing neighbouring aggression (Viblanc *et al.*, 2012). The average HR response was even higher in response to a moving human than it was during logger deployments. However, while a moving human in close proximity caused the HR to be maintained at a higher level (so as to be prepared for immediate action) than during handling (when the birds appeared to have ‘accepted their fate’), integrating the entire procedures demonstrated that handling had an overall greater impact during our experiments.

Given that the birds did not adapt to human presence at the nest, the relative severity of a disturbance stimulus greatly depended on exposure time. We observed unregulated visitors frequently spending more than 20 min at close proximity to penguin nests, which is likely to have resulted in at least as high a response as that measured for 10 min handling the bird. Beale and Monaghan (2004) found that effects of disturbance amplify with increasing numbers of visitors and decrease with distance from the nest. Our experiments involved a single person moving in a calm, steady manner. Conceivably, groups of noisy people with their erratic movements, as regularly observed in the tourist-exposed breeding area, would be associated with higher impact on the birds.

In our case, even when having recovered after human disturbance the birds often remained vigilant, and the RHR was frequently interrupted by alert look events. Furthermore, nest maintenance behaviour after human disturbance was associated with significantly higher HR responses than what we observed naturally. Comparable to Humboldt penguins (Ellenberg *et al.*, 2006), nest maintenance of Yellow-eyed penguins after stressful events can be interpreted as displacement behaviour. Such behaviours depended on individual stress-coping style rather than stimulus, and as such, were a less reliable indicator to quantify the impact of disturbance. Similar to our observations in Snares penguins (Ellenberg *et al.*, 2012), behavioural responses have to be interpreted with caution.

In koalas, RHR may be altered as a result of repeated disturbance events, leading to states of permanent agitation (Ropert-Coudert *et al.*, 2009). Despite lasting agitation of Yellow-eyed penguins after an acute stressor, RHR did not differ between sites exposed to unregulated tourism and those visited for monitoring purposes only, suggesting that the penguins are currently not chronically stressed at the tourism site. This is supported by comparable low baseline corticosterone levels at both breeding sites (Ellenberg *et al.*, 2007).

While a careful ‘wildlife photographer’ spending 20 min in close proximity to a penguin nest may provoke the same integrated response as handling the bird for 10 min, the ­longer-term effects of each is unknown. Capture and handling may be perceived as a predation attempt (Frid and Dill, 2004) and can have prolonged effects on the affected animal. For example, in zoo tigers (*Panthera tigris*), the average immune-reactive cortisol concentrations peaked 3–6 days after transport, despite the tigers being released into a known environment, and needed 9–12 days to return to baseline levels (Dembiec *et al.*, 2004). Likewise, great tits (*Parus major*) in the wild showed sustained mass change for 7 days following handling before mass returned to pre-capture levels (MacLeod and Gosler, 2006). Little is known about longer-term effects of human disturbance on Yellow-eyed penguins. However, it has been shown that stress can have an effect on attention, decision making, or memory of an individual (McEwen and Sapolsky, 1995; Mendl, 1999), and prolonged or frequent exposure to stress can induce higher susceptibility to diseases, reduced fertility, and lower life expectancy (e.g. Siegel, 1980; Wingfield *et al.*, 1997; Sapolsky *et al.*, 2000). We already observe significantly reduced breeding success, lower fledgling weights, and reduced first year survival at frequently visited sites (McClung *et al.*, 2004; Ellenberg *et al.*, 2007).

### Importance of objective information on the effects of disturbance

An animal may perceive disturbance stimuli that we might consider as low impact quite differently. For example, prolonged motionless observation for determination of nest status is generally regarded as being less stressful than a short direct approach to the nest, but in Yellow-eyed penguins has exactly the opposite effect. Likewise, Langkilde and Shine (2006), who measured plasma corticosterone levels in ­lizards, *Eulamprus heatwolei*, Scincidae, found that toe-­clipping for identification, which is often criticized and sometimes banned for ethical reasons, was less stressful than microchip implantation, which caused elevated stress hormone levels that were maintained for 14 days. Toe-clipping was also less stressful than manipulations that may be perceived by humans as trivial, such as housing the animal in an unfamiliar enclosure (Langkilde and Shine, 2006). Thus, it is important to seek objective information on the real effects of human-derived disturbance stimuli.

### Individual differences

The stress response will depend on how dangerous a disturbance stimulus is perceived to be. While we presented average responses here, it is important to bear in mind that individual birds may react differently to the same stimulus. Individual Yellow-eyed penguins differ in their initial stress response and habituation potential to human disturbance depending on their sex, character, and previous experience with humans (Ellenberg *et al.*, 2009). Birds exposed to frequent visitation have not habituated; on the contrary, they appear to be sensitized to humans and showed stronger stimulus-specific HR responses than neighbouring, less-disturbed conspecifics. This is consistent with the elevated hormonal stress response observed in birds exposed to unregulated tourism (Ellenberg *et al.*, 2007). Likewise, Snares penguins (*Eudyptes robustus*) appear to have learned from previous experiences with humans and showed significantly stronger HR responses to experimental human approach following exposure to intrusive research and filming activities (Ellenberg *et al.*, 2012). Thus, penguins at the site exposed to unregulated tourism do not only get disturbed more often, but each disturbance event appears more costly for the birds.

### Management implications

Stressful events may redirect an individual's behaviour towards survival rather than reproduction (Watanuki *et al.*, 1993; Wingfield *et al.*, 1997), and consequently, increase the likelihood of nest abandonment, particularly in long-lived species (Wingfield *et al.*, 1995). For anticipatory management decisions, it is important to determine the relative severity of different disturbance stimuli.

Using HR response to measure disturbance-related impacts, we showed that touching the bird on its chest did not significantly increase the HR response already associated with human proximity. Thus, obtaining data via a quick direct nest-check is not only more reliable but also less disturbing than determining nest status via prolonged observation from a distance. Given that Yellow-eyed penguins do not easily adapt to human proximity, regardless of the whether the disturbance stimuli are of a visual or an acoustic nature, it is important to keep any human activity in their proximity to a minimum. Tourist, research, and management activities need to be carefully evaluated and spatially/temporarily managed to reduce cumulative disturbance impact.
